# A Novel and Cost-Effective Monitoring Approach for Outcomes in an Australian Biodiversity Conservation Incentive Program

**DOI:** 10.1371/journal.pone.0050872

**Published:** 2012-12-06

**Authors:** David B. Lindenmayer, Charles Zammit, Simon J. Attwood, Emma Burns, Claire L. Shepherd, Geoff Kay, Jeff Wood

**Affiliations:** 1 Fenner School of Environment and Society, The Australian National University, Canberra, Australian Capital Territory, Australia; 2 Australian Research Council Centre of Excellence for Environmental Decisions, The Australian National University, Canberra, Australian Capital Territory, Australia; 3 National Environmental Research Program, The Australian National University, Canberra, Australian Capital Territory, Australia; 4 Biodiversity Conservation Branch, Australian Government Department of Sustainability, Environment, Water, Population and Communities, Canberra, Australian Capital Territory, Australia; The Australian National University, Australia

## Abstract

We report on the design and implementation of ecological monitoring for an Australian biodiversity conservation incentive scheme – the Environmental Stewardship Program. The Program uses competitive auctions to contract individual land managers for up to 15 years to conserve matters of National Environmental Significance (with an initial priority on nationally threatened ecological communities). The ecological monitoring was explicitly aligned with the Program’s policy objective and desired outcomes and was applied to the Program’s initial Project which targeted the critically endangered White Box-Yellow Box-Blakely's Red Gum Grassy Woodland and Derived Native Grassland ecological community in south eastern Australia. These woodlands have been reduced to <3% of their original extent and persist mostly as small remnants of variable condition on private farmland. We established monitoring sites on 153 farms located over 172,232 sq km. On each farm we established a monitoring site within the woodland patch funded for management and, wherever possible, a matched control site. The monitoring has entailed gathering data on vegetation condition, reptiles and birds. We also gathered data on the costs of experimental design, site establishment, field survey, and data analysis. The costs of monitoring are approximately 8.5% of the Program’s investment in the first four years and hence are in broad accord with the general rule of thumb that 5–10% of a program’s funding should be invested in monitoring. Once initial monitoring and site benchmarking are completed we propose to implement a novel rotating sampling approach that will maintain scientific integrity while achieving an annual cost-efficiency of up to 23%. We discuss useful lessons relevant to other monitoring programs where there is a need to provide managers with reliable early evidence of program effectiveness and to demonstrate opportunities for cost-efficiencies.

## Introduction

The importance of incorporating biodiversity conservation into sustainable agricultural production systems is now recognized around the world [1], including in North America [2], Central America, South America [3], Africa [4], Europe [5] and Australia [6], [7].

One emerging and increasingly popular approach to addressing biodiversity conservation in agricultural landscapes has been to create market-based incentive schemes that pay private land managers (often farmers) to undertake specific conservation actions as part of a funding agreement [8]–[14]. Some of these schemes are large in scale and well-funded. For instance, in 2009 in the United Kingdom, £497 million was spent on agri-environment schemes [15], while the EU contribution to agri-environment schemes for 2001–2003 was approximately €2 billion per year [16].

Despite this considerable expenditure, these schemes have been criticized (e.g. [8], [17]–[19]) for often not adequately monitoring environmental outcomes [20]. Such criticisms are not restricted to Europe. Inadequate ecological monitoring has made it almost impossible to determine the effectiveness of the $US15 billon river restoration programs in the USA [21] and an equivalent (but smaller) multi-million dollar river restoration program in Australia [22]. Also in Australia, deficiencies of ecological monitoring have been highlighted in the multi-billion dollar Natural Heritage Trust and National Salinity Action Plan initiatives intended to support environmental management, including in agricultural areas [23]–[25]. A lack of past investment in program monitoring and reporting means that the effectiveness of public environmental expenditure cannot readily be demonstrated [26], [27]. Furthermore, this shortcoming reduces opportunities for determining effectiveness of management actions, identifying new issues for research, and building land manager understanding and capacity [25], [28].

Quantifying the outcomes of both public and private investments in conservation programs is an enduring challenge for many reasons [28], [29]. Historically, conservation policy outcomes have been couched in broad, aspirational terms making their measurement problematic. Alongside this, public funding for biodiversity conservation is inevitably constrained by competing budget priorities across government and there is often reluctance to direct scarce funds away from on-the-ground interventions that aim to deliver improvements for biodiversity. Moreover, funding cycles for conservation projects are typically short (<3 years), so the likelihood of achieving detectable ecological benefits is low.

In addition to these policy constraints, the funding and reward priorities for academic ecologists have generally mitigated against research funding being directed towards developing cost-effective and reliable monitoring systems for public policy assessments. This is in contrast to medical and social research where substantial public funds are invested in large scale and long-term monitoring programs. For these and other reasons, conservation programs have consistently under-invested in long-term monitoring and reporting [23], [30].

In this paper, we describe a relatively recent Australian example of an incentive program that incorporated monitoring as an essential feature of its implementation [14], [31]. The Australian Government’s Environmental Stewardship Program (hereafter termed the “Program”) aims to maintain and improve the condition and extent of targeted Matters of National Environmental Significance on private land by using market-based approaches [31]–[33]. Matters of National Environmental Significance are listed by the Australian Government under the *Environment Protection and Biodiversity Conservation Act 1999*. The Program contained several innovations on previous market-based incentive schemes in Australia. Three of these were:

- A commitment to annual funding for landholders up to 15 years.
- Annual performance-based payments to ensure compliance.
- The development of fit-for-purpose ecological and social monitoring [14], [34].

The Australian Government contracted The Australian National University (ANU) to develop and implement ecological monitoring for the Program’s initial Project. This Project targeted the critically endangered White Box-Yellow Box-Blakely's Red Gum Grassy Woodland and Derived Native Grassland (BGGW) ecological community (hereafter known as the Project). These woodlands occupy the wheat/sheep belt of temperate eastern Australia and have been extensively cleared for agriculture, resulting in less than 3% remaining, often in small and degraded remnants predominantly on private land [14]. The Project sought to specify clear and achievable biodiversity outcomes, provide cost-efficient long-term investments, and establish a multi-layered monitoring and compliance strategy [35].

The monitoring contract was to design and implement four years of monitoring and also to outline how sampling might be extended over the 15 year duration of the land manager contracts (up to June 2023). Here we describe the approach, initial results, and learning experiences from this monitoring. We document the initial costs of the various phases of design and implementation and provide a forecast of future costs for the monitoring project. We also demonstrate the efficiency and benefits of a proposed rotating sampling approach to monitoring (*sensu* [36]) that reduces survey costs over time while still maintaining sufficient power to detect relevant ecological changes that meet policy objectives. This approach involves a subset of sites being monitored in any given year and for 50–70% of all sampled sites to remain the same from one year to the next. This contrasts with traditional approaches of resampling a fixed set of monitoring sites in a given time period (e.g. annually).

We suggest the approach taken to establish and implement the ecological monitoring, including the efficiencies of a rotating sampling approach once initial benchmark surveys have been completed, will have broad relevance to other conservation programs. In particular, our approach will be valuable where there is a need to provide reliable early evidence of program effectiveness.

## Methods

### Ethics Statement

Our studies were observational investigations and no plants or animals were harmed. The monitoring project was conducted in accordance with the requirements of permit F.ES.04.10 issued by the Animal Experimentation Ethics Committee of The Australian National University. We also obtained a permit under the Queensland Nature Conservation (Administration) Regulation 2006 (no. WISP084601910) and a scientific research license issued by the New South Wales National Parks and Wildlife Service (no. 13174). No specific permits were required for access to private farms as the owners had established access relationships with the Program.

### The Design of the Ecological Monitoring Project

**Figure 1:**
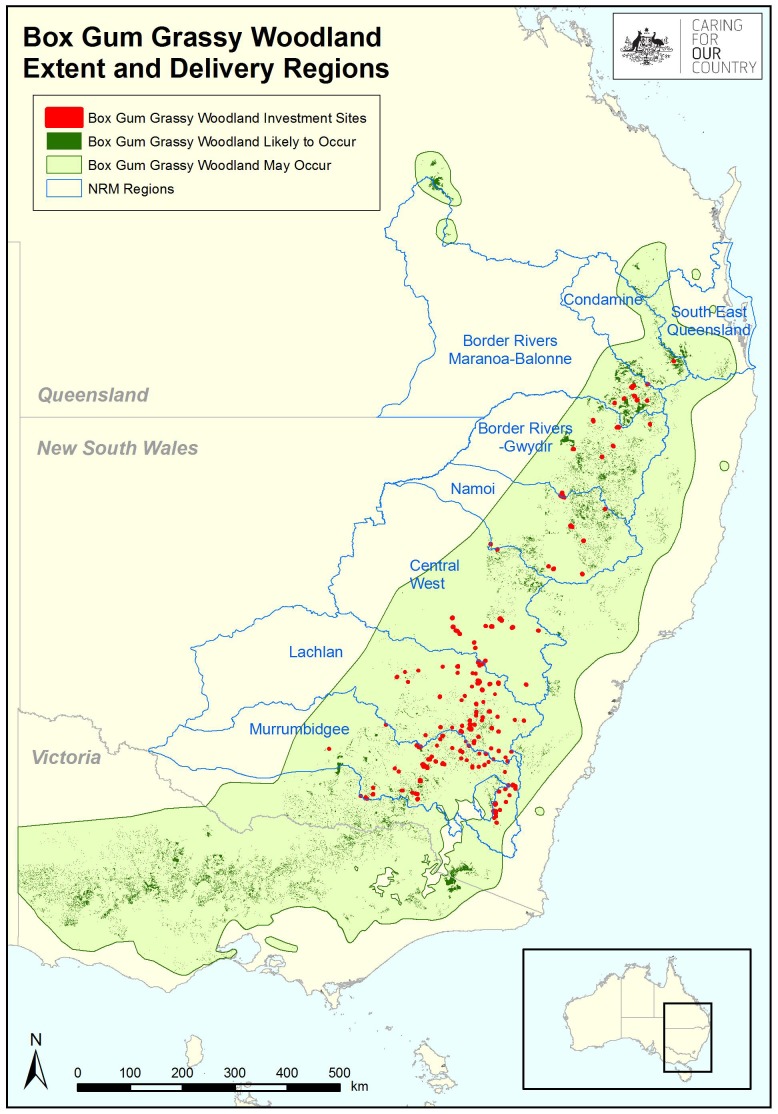
Locations of 153 stewardship sites from rounds 1–4 of the BGGW Project. The blue polygons denote the location of Natural Resource Management (NRM) regions that were established to support regional scale planning and investment in natural resource management and biodiversity conservation on public and private land.

The design of the ecological monitoring commenced in 2009–2010. The Australian Government had by then contracted 201 land managers for up to 15 years to conserve 26,000 ha of box gum grassy woodland on 153 farms from southern New South Wales to southern Queensland. These farms covered an area of 172,232 sq km encompassing seven Natural Resource Management (NRM) regions, making it one of the most ambitious, large-scale monitoring programs implemented in Australia (Figure 1).

A requirement of the Program was for all sites on contracted farms to be available for ecological monitoring and for all participating land managers to be informed of the monitoring results. The broad aims of the ecological monitoring were: **(1)** to determine if funded management actions are improving the condition and extent of the woodland over time; and **(2)** to determine whether measured changes in vegetation condition/extent influence the distribution and abundance of particular groups of biota. In addition to the specific monitoring of the funded sites, the Australian Government also supported an experiment to establish the efficacy of the prescribed livestock grazing management regime used in the Project (see Supporting Information S1).

We established a monitoring site within a funded woodland patch (hereafter termed a stewardship site) on all 153 farms. We then established a control site on 115 farms with paired control and stewardship sites matched on the basis of vegetation type, vegetation condition, and other characteristics such as landform, patch size and patch connectivity. It was important to ensure the matched control sites were located on the same farm as the stewardship sites. This was because previous woodland research work showed that farm-level management affects biodiversity [37]. On each farm, stewardship and control sites were located ∼1.5–2 km apart to reduce the potential for spatial dependence.

Control sites were unaffected by the Project and were part of ‘business-as-usual’ farm management practices (e.g. grazing, invasive plant and animal control). Establishing matched stewardship and control sites will enable us to determine if the observed changes on a stewardship site are due to management intervention rather than other factors, including those operating at larger spatial scales (e.g. climate conditions).

On 38 of the 153 farms it was not possible to identify a control site. This was due to the absence of the target woodland community (other than the patch managed under the Project), or too great a difference in the variables used to match sites.

### A Novel and Non-standard, Overlapping and Rotating Statistical Design to Guide Field Surveys

Our monitoring design comprised 268 monitoring sites (153 stewardship and 115 control sites). In the early years of the monitoring, field surveys of plants, birds and reptiles will be completed at all 268 sites. We will then use a novel and non-standard, overlapping and rotating statistical design [36] to guide monitoring in a subset of ∼50% of the 268 sites in any given year. This design will allow for 50–70% of all sampled sites to remain the same from one year to the next. Every 5^th^ year, a full census of all 268 monitoring sites will be conducted. This overlapping and rotating statistical design was developed for a long-term monitoring program of seabirds in the Coral Sea [36] and then further refined in a monitoring program for arboreal marsupials in the wet forests of Victoria, south-eastern Australia [38].

### Site Establishment and Vegetation Surveys

**Figure 2:**
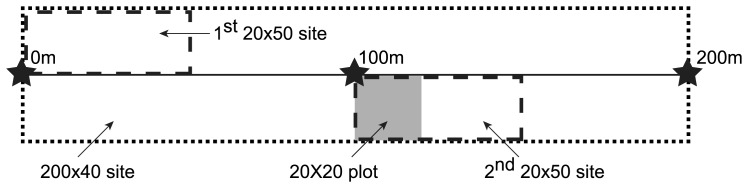
Schematic diagram of vegetation sampling on Stewardship and spatial control sites.

At each of our 268 sites, we established a 200 m long permanent transect with steel pickets at the 0 m, 100 m and 200 m points. Thus, the ‘site’ was the unit of measurement for all locations in the overall study. We conducted vegetation surveys along the length of each transect to estimate the levels of vegetation cover. In addition, we measured vascular plant species richness in a 20 m×20 m plot at the 100 m point along the transect and established two 50 m×20 m plots to gather data on vegetation structure (Figure 2).

Our vegetation monitoring served four key purposes: **(1)** benchmarking of condition prior to intervention, **(2)** quantifying changes in condition over time, **(3)** determining progress toward policy goals, and **(4)** provision of a suite of covariates for use in developing statistical models of the relationships between vegetation condition, treatment intervention and biotic responses (e.g. for fauna – see below). Supporting Information S2 illustrates the links between variables measured and the broader suite of desired Program outcomes.

### Fauna Surveys

Although the Project’s primary policy objective is to maintain and improve the condition and extent of the target woodland, there was merit in also monitoring some faunal groups. This was because changes in vegetation condition can influence changes in faunal assemblages [39], [40]. Moreover, the Program’s Strategic Framework includes “*improved habitat*” and “*improvement in…function of ecological communities*” as desired outcomes (see Supporting Information S2). Therefore, monitoring faunal responses provides an indication of the extent to which these outcomes have been met [31].

We selected two groups of fauna for monitoring – birds and reptiles. Past work has indicated that some elements of the bird biota respond strongly to various attributes of vegetation condition like native ground cover, the presence of understorey, and the abundance of fallen timber (e.g. [39], [41]). Furthermore, many species of birds in grassy woodland ecosystems are of conservation concern because they may be declining [42], in part due to a range of threats that the Project seeks to mitigate. These include clearance of native vegetation, livestock over-grazing, and exotic plant invasion [43], [44].

Our bird counting protocols comprised repeated 5 minute point interval counts (*sensu* [45]) at the 0 m, 100 m and 200 m points along the transects. In any given year, each site is counted twice by a different observer on a different day to limit day and observer effects (see [46], [47]). All birds are counted within 100 m of a given transect point. This approach generates reliable presence-absence and detection frequency data.

We conducted surveys of woodland reptiles because most species operate at smaller spatial scales and respond to different structural attributes than most birds. Our past work also indicated that some elements of the reptile biota respond strongly to various attributes of vegetation condition, such as the cover of native grass tussocks (e.g. [37]). Reptiles also can be negatively affected by the removal of course woody debris and bush rock [48], [49], both of which are threats that the Project seeks to address. We employed two reptile monitoring protocols: **(1)** establishing artificial substrates (tiles, corrugated sheet metal and wooden sleepers) at the 0 m, 100 m and 200 m points along each transect, and **(2)** completing a 30 minute time-constrained active search.

### Statistical Analyses

For the preliminary and illustrative results that we present in this paper, we calculated species richness for plants, birds and reptiles and fitted Hierarchical Generalized Linear Models (HGLMs) [50] using a quasi-Poisson model with a logarithmic link. We assumed that farm effects were random with a gamma distribution and a logarithmic link function. The fixed effects were stewardship versus control treatment and NRM region. Thus, we assumed that if the species richness for stewardship sites differed from that for control sites, the ratio would be similar for all NRM regions.

### Costs of Ecological Monitoring

We compiled information on the key steps involved in establishing the monitoring including the development of the survey methods, the identification and establishment of field sites, surveys of vegetation, reptiles and birds, and data analysis and reporting. We then estimated the costs associated with each of these steps through estimating the amount of time, effort and equipment required to complete each stage. Finally, we made budget projections to June 2023 to cover the period of land manager contracts under the Project. The projection includes two cycles of 5 years (4 years of rotating sampling followed by a full census of all 268 sites).

## Results

### Vegetation

We categorized vegetation data into several aggregated vegetation components including overstorey, midstorey, groundcover (perennial grasses), groundcover (forbs), groundcover (sub-shrubs), and groundcover (other).

**Figure 3:**
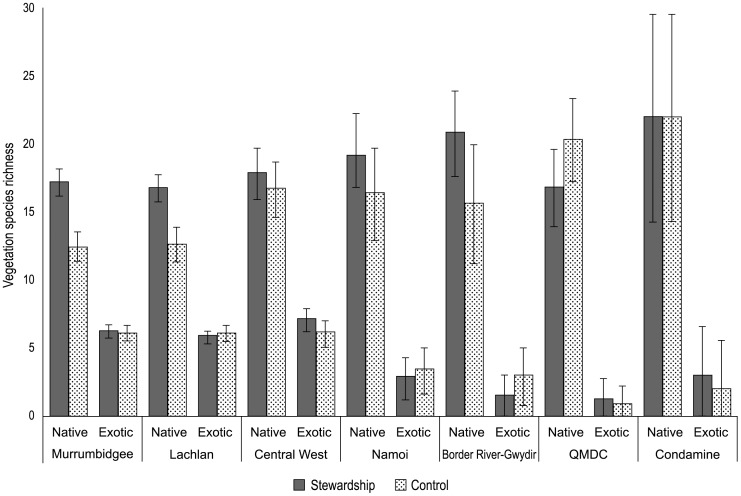
Average species richness across sites for plants.

We found that within NRM regions, native plant species richness varied significantly between stewardship and control sites (F_1,246_ = 13.5; P<0.001) – a result consistent with findings from across the entire Project area (Figure 3). We found no significant among-NRM region differences (F_6,246_ = 1.40; P = 0.210) in native plant species richness on stewardship and control sites. However, when separated into vegetation components, we found significant differences among NRM regions in the species richness of native midstorey plants (F_6, 246_ = 2.85; P = 0.011), perennial grasses (F_6, 246_ = 2.94; P = 0.009), sub-shrubs (F_6, 246_ = 3.05; P = 0.007) and other (e.g. rushes/sedges) (F_6, 246_ = 2.93; P = 0.009).

Within NRM regions, we found that exotic plant species richness did not differ significantly between stewardship and control sites (F_6,246_ = 0.02; P = 0.896). However, exotic plant species richness was significantly different among NRM regions (F_6, 246_ = 6.79; P<0.001) and was highest in the Murrumbidgee, Lachlan and Central West NRM regions (Figure 3).

### Birds and Reptiles

**Figure 4:**
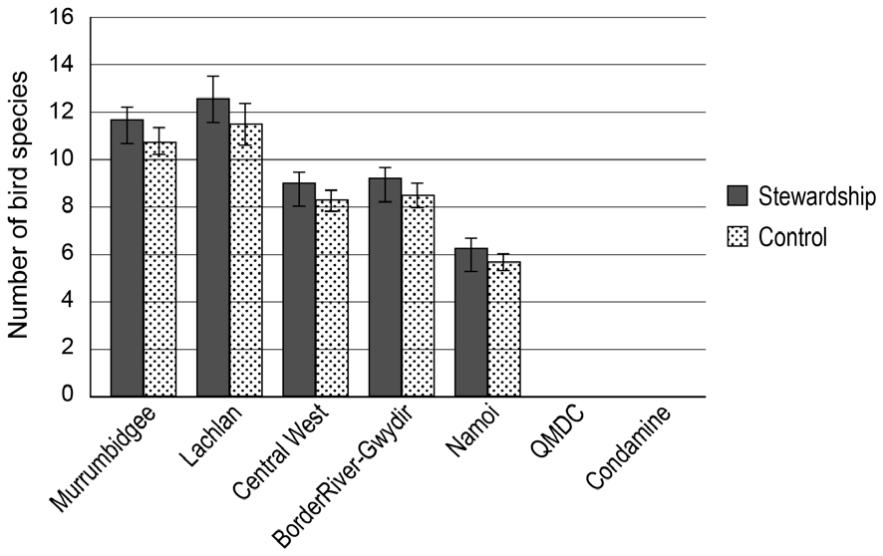
Average species richness across sites for birds. Two NRM regions were not sampled for birds because flooding precluded access to field sites.

Species richness for birds was significantly greater on stewardship than control sites (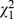 = 4.1; P = 0.042). When separated by NRM region, average bird species richness varied significantly among all NRM regions (F_4,219_ = 3.54; P = 0.008) and was highest in the Murrumbidgee and Lachlan regions (Figure 4).

**Figure 5:**
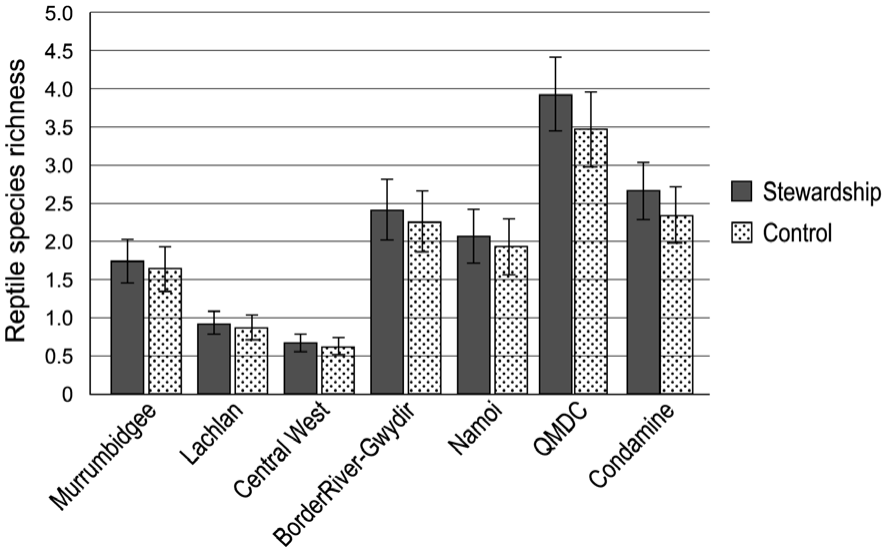
Average species richness across sites for reptiles.

We found that across the Project area, and within NRM regions, reptile species richness did not differ significantly between stewardship and control sites. However, species richness did vary significantly among NRM regions (F_6,240_ = 10.10; P<0.001). NRM regions in the north supported higher average richness per site than NRM regions in the south (Figure 5).

### Effort and Costs of Establishing and then Maintaining Ecological Monitoring

We summarize the resources and costs of establishing and then maintaining the ecological monitoring in the remainder of this section, as well as outline the rationale for expenditure in Supporting Information S3. We present a budget in Table 1 that summarizes actual costs over the first 3 years and forecasts future expenditure for the ecological monitoring. We do not provide a full explanation of the budget here but make observations that are of interest to those aiming to establish an ecological monitoring system for a conservation program.

The budget in Table 1 covers the four-year funded establishment phase followed by two 5-year monitoring cycles, and totals around $6.57 m assuming no variations year to year apart from the proposed rotating sampling and purchase of vehicles every three years. Under this budget, 70% is for staff, 25% is for operational expenses (including field travel), and 5% for vehicle purchases.

**Table 1:** Costs of the monitoring program for the Box Gum Grassy Woodlands Project within the Environmental Stewardship Program.

			Year 1	Year 2	Year 3	Year 4	Year 5	Year 6	Year 7	Year 8	Year 9	Year 10	Year 11	Year 12	Year 13	Year 14
			2009/10	2010/11	20011/12	2012/13	2013/14	20014/15	2015/16	2016/17	2017/18	2018/19	2019/20	2020/21	2021/22	2022/23
			Start-up	Full census	Full census	Half sample	Half sample	Half sample	Half sample	Full census	Half sample	Half sample	Half sample	Half sample	Full census	Half sample
Project management		Operational^1^	27,300	68,012	61,364	54,907	60,369	57,306	58,791	72,558	61,311	62,348	67,919	65,002	76,793	71,752
(inc grazing experiment)		Field staff^2^	182,818	188,710	174,947	155,920	160,597	165,415	170,378	175,489	180,754	186,176	191,762	197,514	203,440	209,543
		Capital^3^	0	89,690	0	0	50,000	0	0	50,000	0	0	50,000	0	0	50,000
		Subtotal	210,118	346,412	236,311	210,827	270,966	222,721	229,168	298,047	242,065	248,524	309,681	262,516	280,232	331,295
Design start-up		Operational	0	0	400	0	400	0	200	0	200	0	200	0	200	0
and review		Field staff	88,881	0	3,000	0	3,000	0	3,000	0	3,000	0	3,000	0	3,000	0
		Subtotal	88,881	0	3,400	0	3,400	0	3,200	0	3,200	0	3,200	0	3,200	0
Site set-up		Travel^4^	85,857	19,880	4,000	4,000	4,000	4,000	4,000	4,000	4,000	4,000	4,000	4,000	4,000	4,000
and maintenance		Field staff	123,844	34,527	0	4,200	4,326	4,456	4,589	4,727	4,869	5,015	5,165	5,320	5,480	5,644
		Subtotal	209,701	54,407	4,000	8,200	8,326	8,456	8,589	8,727	8,869	9,015	9,165	9,320	9,480	9,644
Vegetation		Travel^5^	111,873	17,136	17,650	11,250	11,588	11,935	12,293	25,324	13,042	13,433	13,836	14,251	29,357	15,119
		Field staff	54,806	55,362	57,023	29,948	30,846	31,772	32,725	67,414	34,718	35,760	36,832	37,937	78,151	40,248
		Subtotal	166,679	72,498	74,673	41,198	42,434	43,707	45,018	92,738	47,760	49,193	50,668	52,188	107,508	55,367
Reptiles		Travel	0	5,712	5,883	4,200	4,326	4,456	4,589	9,454	4,869	5,015	5,165	5,320	10,960	5,644
		Field staff	0	18,454	19,008	11,181	11,516	11,862	12,217	25,168	12,961	13,350	13,751	14,163	29,176	15,026
		Subtotal	0	24,166	24,891	15,381	15,842	16,317	16,807	34,622	17,830	18,365	18,916	19,484	40,136	20,670
Birds		Travel	0	15,232	15,689	9,975	10,274	10,582	10,900	22,454	11,564	11,911	12,268	12,636	26,030	13,406
		Field staff	0	49,210	50,687	26,554	27,351	28,171	29,016	59,773	30,783	31,707	32,658	33,638	69,294	35,686
		Subtotal	0	64,442	66,376	36,529	37,625	38,754	39,916	82,227	42,347	43,617	44,926	46,274	95,324	49,092
Statistical support and data analysis			34,652	13,362	13,763	17,363	17,884	18,420	18,973	24,428	20,129	20,732	21,354	21,995	28,319	23,335
Total			**710,031**	**575,287**	**423,413**	**329,498**	**396,477**	**348,375**	**361,672**	**540,789**	**382,199**	**389,447**	**457,911**	**411,778**	**564,200**	**489,403**
**Every year full census**			Start-up	Full census	Full census	Full census	Full census	Full census	Full census	Full census	Full census	Full census	Full census	Full census	Full census	Full census
Total			710,031	575,287	423,413	430,054	500,050	455,055	471,552	540,789	498,772	509,516	581,583	539,159	564,200	624,542

Note: A 3% p.a. inflation rate has been applied.

1 Operational includes University overhead, annual vehicle registration, insurance and fuel, telephones, field equipment.

2 Staff cost after Year 2 is 1.0 FTE research officer and 1.0 FTE assistant, reduced in this line item by the amount attributed to their participation in field surveys.

3 Year 2 purchase 2 new 4WDs. Sell and replace 2×4WDs every 3rd year.

4 Includes periodic use of up to 5 4WD vehicles in Year 1.

5 Comprises per diem accommodation and food for survey period.

All costs are in Australian dollars and include in-kind co-contributions from ANU in addition to Australian Government funds. Years after 2012–2013 are budget projections for future monitoring in the Project for both: (a) a typical sample year (i.e. 4 out of every 5 years of field surveys) in which a subset of (50%) of the field sites are surveyed for birds, reptiles and vegetation as part of the rotating statistical design, and (2) a full census year (one in every 5 years) in which all 268 field sites are surveyed. For comparative purposes, a costing for a program of full census every year is given at the bottom of the table.

**Figure 6:**
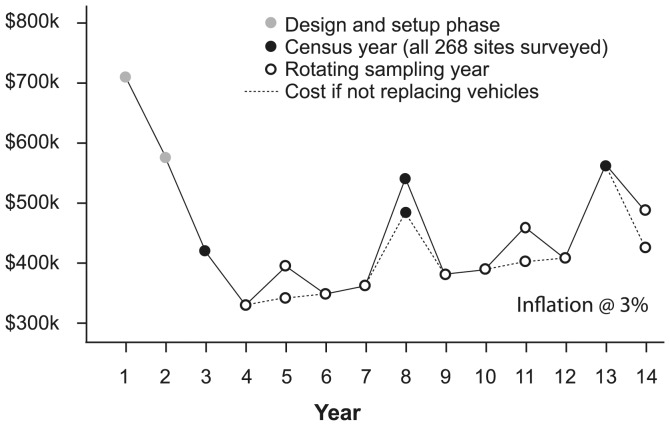
Approximate budget costs (to Year 3) and projected monitoring costs (Years 4–14) for the monitoring for the Box Gum Grassy Woodlands Project within the Environmental Stewardship Program. Detailed budget figures are given in Table 1. After the establishment phase, there is a five year cycle consisting of 4 years of partial sampling in which a subset of sites are surveyed under a rotating sampling regime (*sensu* [36]), followed by one year when all 268 sites in the monitoring program are surveyed. Estimated costs are elevated every three years when 4WD vehicles are replaced.

Following the full establishment and implementation of the monitoring (between 2009 and 2012), we propose to use a rotating sub-sampling approach to select a subset of ∼50% of the 268 sites for monitoring in four of every five years. Every 5^th^ year there will be a full census of all 268 sites; that is, all sites will be monitored. Using this approach, the typical annual budget for the majority (4 of 5) years, when a half sample is conducted, can be reduced by about 23% of the equivalent budget for a full sample year (Table 1; Figure 6). For example, the budget for the ecological monitoring can be reduced to ∼$A329,500 in 2012–2013, compared to $430,000 if a full sample was conducted in that year (Table 1). Over each 5 year cycle (which will include one full census year, and one or two years carrying an additional budget for vehicle replacement), the cost saving would be around 18%.

Table 1 is the direct financial costs of the monitoring, we have not included a wide-range of in-kind costs because these are difficult to calculate and it is difficult to reasonably bound these estimates. Costs of this type would include the extensive expertise and goodwill generated from two decades of working in woodlands prior to this monitoring, and administrative costs borne by The Australian National University and the Australian Government.

## Discussion

There is now a well-established and growing literature on the importance of biodiversity monitoring and reporting based on modern ecological theory and practice [26], [29], [51], [52]. However, there remains an ongoing challenge for policy makers, scientists and managers on how best to incorporate this scientific expertise into conservation policies and programs and how to shape policy-useful research [53]–[56].

The Environmental Stewardship Program has fostered effective linkages between policy makers, private land managers and researchers. This is because the design of the Box Gum Grassy Woodland Project has explicit investment prioritization underpinned by a purpose-built metric, and has established a hierarchical monitoring and compliance system that incorporates effective ecological monitoring as outlined here [14].

The monitoring we have described here was designed to assess progress in achieving the Program’s policy outcomes and its implementation. It has produced some important lessons that we contend can be of use to policy makers, biodiversity managers and applied ecologists. We outline four of these lessons in the remainder of this paper.

### Lesson #1: There are Important Inter-relationships between the Costs of Monitoring Programs, and Maintaining their Cost-efficiency and Scientific Integrity Over Time

Over time, various ‘rules of thumb’ have evolved among researchers and program managers that suggest monitoring and reporting budgets should be around 5–10% of program budget [30], [57]. The Project has invested approximately $A70.53 million for specific conservation outcomes to be achieved on more than 26,000 hectares over 15 years. The monitoring budget for the period to June 2013 is $A2.038 million, or 8.49% of the on-ground investment of ∼$A24 million to that date.

If monitoring using the rotational model was implemented until June 2023, 9.05% of the total Project cost of $70.53 million investment would be devoted to monitoring. This is consistent with, but close to the upper limit of, the monitoring budget ‘rules of thumb’ [58]. Notably, the first two establishment years for the monitoring were comparatively expensive (Table 1), primarily because of the spatial scale of the Project as well as the isolation of many farms (see Figure 1).

Ecological monitoring can sometimes appear to be expensive and there can be challenges in sustaining the budget for it. Overall costs and cost-effectiveness are therefore key issues. In the case of the monitoring we report here, a substantial expense is salary and the travel costs of staff visiting the many farms distributed across the large geographic spread of the Project.

To reduce costs, fewer farms could be visited, or at each farm fewer measurements could be taken, or farms could be visited less frequently. There are a number of ways to adopt these options and we briefly outline three realistic ones below, with a longer discussion of why we favor the third one which is based on a rotating sampling approach.

One option was to reduce the number of farms visited, with the more remote farms in the northern part of the study area (north-eastern New South Wales and south-east Queensland; see Figure 1) being the most obvious ones (on the basis of cost) to cease sampling. This would then entail a traditional site revisit approach with the reduced number of remaining (southern) sites being re-surveyed every year. This option was not considered further because: **(1)** Deletion of farms in the north of the study region would have substantially reduced the ability to make spatial inferences about the impacts of the management regimes employed under the Project that could be drawn from the ecological monitoring. **(2)** There are contractual requirements in the Project to monitor all farms. And **(3)** A design with no northern sites may have been perceived as too southern-focused which may have had a detrimental impact on Program engagement with northern landholders.

A second option to reduce costs was to focus monitoring on vegetation condition and drop other groups such as birds and/or reptiles. Thus, all sites would be resurveyed every year but only one taxon – plants – would be monitored. This option would meet the primary policy objective of the Program to improve vegetation condition but also has a number of deficiencies and so was not favored. A major deficiency was that solely targeting plants would not allow the Program to assess its broader set of desired outcomes including those related to enhancing habitat and ecological community function [31]. In our current design, this is achieved through quantifying relationships between changes in vegetation condition and its impacts on birds and reptiles. Over time, the surrogacy value of monitoring only vegetation condition can be more rigorously tested from the additional data that are collected. Moreover, a requirement of the monitoring was to provide timely feedback on the effects of altered management practices to individual landholders. In our experience, birds are the group for which landholders have the greatest interest in understanding change, whereas reptiles are the group found in other work to exhibit the fastest rate of response to management intervention.

A third option to reduce costs was to visit farms less frequently. This can be achieved by employing an overlapping and rotating statistical design and subsample from the total pool of all 268 monitoring sites in any given year. This was the preferred option for a suite of reasons beyond the ability to reduce costs (by ∼23%) in most years. The approach ensures that all groups (plants, birds and reptiles) continue to be monitored and hence the advantages of doing this are maintained such as: **(1)** Linking changes in vegetation condition to changes in animal populations. And **(2)** The monitoring is inclusive of landholders from all natural resource management regions and it includes animals of the greatest interest to most landholders (typically birds). Such inclusiveness also aids communication of results to landholders and increases opportunities to build social capacity for improved management practices on the ground. In addition, the rotating sampling approach avoids a reduction of statistical integrity. For example, the retention of the full pool of 268 sites means that the broad spatial scope of inferences from the monitoring can be maintained. This advantage is particularly pertinent to the Project which encompasses a large number of sites distributed across a very large area (see Figure 1). There are other statistical advantages of non-standard, overlapping, rotating designs for monitoring and these are discussed by Welsh et al. [36].

In summary, we suggest that a powerful lesson from our approach to ecological monitoring in the Project was that it is possible to reduce survey costs while still maintaining sufficient power to detect change and meeting key policy and other objectives.

### Lesson #2: It is Important to Design Fit-for-purpose Ecological Monitoring to Match the Specific Conservation Policy Objectives and Program Size

The ecological monitoring we have described here is fit-for-purpose. That is, specific details of its design and implementation are tailored to the explicit objective and desired outcomes of the Environmental Stewardship Program’s Box Gum Grassy Woodland Project [31] (Supporting Information S2). This level of specification arose through close collaboration between researchers and policy makers within the Environmental Stewardship Program. Resulting particular specifications were:

- To sample all contracted farms from the first four tender rounds to establish an ecological benchmark at the start of the Program.
- To include matched control sites on farms whenever feasible.
- To include the monitoring of birds and reptiles alongside vegetation to provide a more complete picture of the ecological dynamics of nationally critically endangered temperate woodlands over time.
- To incorporate experimental manipulations that allows more specific testing of management effectiveness (Supporting Information S1).
- To recognize the value of close engagement with individual farmers and to create opportunities for information sharing and capacity building.

All of these specifications had cost implications that extended the monitoring beyond what might be considered an “adequate” investment to meet the primary objectives of the Program. These were considered acceptable given: **(1)** The national scope of the Program. **(2)** The significant budget allocation provided to design and implement an innovative, market-based approach to the conservation of Matters of National Environmental Significance. And **(3)** The recognition of the need for robust science to underpin and inform investment decisions. However, this level of specificity also means that the precise protocols and details of this design may not be appropriate for uncritical application in other programs with different desired outcomes, and types and scales of intervention/s. Nevertheless, the broadly applicable principle is that conservation programs with clearly stated and measurable objectives allow specific monitoring protocols to be designed. Shared knowledge and expertise from both policy makers and researchers is needed to achieve this.

### Lesson #3: There is a Need to Benchmark Condition, and Implement and Analyze Monitoring Data as soon as Possible After Program Commencement

We suggest that the initial benchmarking and ongoing monitoring of change needs to be incorporated into environmental program design as a foundational activity. This is in contrast to many initiatives where monitoring is undertaken retrospectively and often well after the implementation of large programs (e.g. [30], [59]). The late onset of monitoring may create major problems such as a failure to document pre-treatment conditions (e.g. see Figures 3,4,5) and the subsequent misinterpretation of effects (such as differences between treatment and control sites) being a product of intervention. In addition, thinking of how to monitor for outcomes while designing a program of expenditure can assist in designing a program that is actually ‘measurable’.

Our experience from this monitoring indicates it is critical to analyze datasets early and repeatedly in the data collection process. This is to ensure that problems can be identified, rectified or otherwise addressed in a timely manner. For example, early analyses of our first year of field data revealed statistically significant differences between stewardship and control sites for plant and bird species richness (Figures 3, 4). Hence, these groups of sites have different starting points prior to commencing the management interventions at the stewardship sites. Statistical analysis of the longitudinal data generated by the monitoring will focus on changes from initial values rather than absolute values. We envisage initially fitting time series models such as autoregressive models to the data, but the precise nature of the models will be adapted after closer examination of the data. Differences between stewardship and control sites are small relative to the differences among NRM regions, and the differences between stewardship and control sites are much smaller than the anticipated differences after the Program has been in place for some years.

A second reason to analyze datasets early is that it can help determine if a program’s design is well targeted and/or whether additional or alternative monitoring designs are warranted. For example, although not a primary objective of the monitoring, we recorded four nationally threatened bird and reptile species and 12 other species of birds of conservation concern [*sensu* 41]. Thus, it is clear that the sites that have been funded are of considerable conservation value, as might be expected from woodlands listed as critically endangered. Therefore, management actions will take place in areas where threatened and declining species do occur, the value of which (in an incentive program context) was highlighted by Whittingham [60]. This is important because the results of studies of agri-environment schemes elsewhere in the world have suggested that management interventions benefit common species but have limited positive impact on declining or threatened species [61,62[; although see [63]. Unlike the Environmental Stewardship Program however, such schemes do not target the most at-risk biodiversity, but rather focus on ecological improvements to habitats more generally, with differential impacts on taxa [64]. It is too early to know if the Project is having a positive impact on threatened and declining species, but we know from the outset that species of conservation concern are present on some sites.

A third reason to monitor and analyze data early is to provide land managers with up-to-date and understandable ecological information about the biodiversity on their farms, and over time, how their management interventions are working. On this basis, two popular books were widely circulated to communicate key conservation and management messages [65], [66] to land managers participating in the Project.

### Lesson #4: Effective Links are Critical between Science-literate Policy Makers and Policy-literate Scientists

The Environmental Stewardship Program’s Project has been successfully designed and implemented, in large part, because of collaboration between researchers with previous experience in conservation policy and policy makers with expertise in ecological research. These complementary past experiences are critical for building a shared literacy for what is needed and what is possible [67]. However, as others have noted, the linkages between science and policy and the effective uptake of scientific knowledge into policy remains complex and contested [68], [69]. Accordingly, we do not presume that this lesson, in itself, will resolve many of these complexities, but a shared literacy should improve understanding [70] and increase the effectiveness of dialogue and other forms of collaboration.

While it would be ideal for collaborative partnerships between individual scientists and policy makers to persist over long periods, this is unrealistic. What is clear from our experience is that both policy and research cultures do not sufficiently recognize and foster these sorts of professional relationships [26]. For example, academic research performance is based disproportionately on publications in internationally recognized peer-reviewed journals. Research collaborations with government policy areas, where partnerships such as the monitoring will provide significant and direct public benefits independently of individual publications, are barely recognized. Policy makers, in turn, are public servants who work for elected governments and who often have complex and demanding short-term priorities that do not always lend themselves to enduring partnership investments with research organizations [67], [68]. However, the decision by the Australian Government to create a four year $70 million National Environmental Research Program with an explicit focus on policy-relevant conservation research may go some way towards bringing the policy and research cultures together (http://www.environment.gov.au/biodiversity/science/nerp/about.html). Creating complementary institutional incentives that build research literacy among policy makers and policy literacy among researchers is feasible and worthwhile [71].

### Conclusions

The ecological monitoring being undertaken for the Environmental Stewardship Program’s Box Gum Grassy Woodland Project represents a useful example of rigorously designed, fit-for-purpose monitoring which was established as the conservation program commenced. The initial investment of ∼8.5% of the Project costs over the first four years is within the general rule-of-thumb of 5–10% for investments in monitoring [30], [57]. By using a rotating sampling design, we have demonstrated that it is possible to keep the costs of the whole ecological monitoring program within this rule-of-thumb while still maintaining sufficient power to detect change, as well as meeting policy objectives.

Ecological monitoring for the Project has generated useful lessons for ecological researchers, policy makers and conservation managers working in agricultural landscapes. Among the most important, in our view, is the value to individuals and their organizations of documenting the costs of fit-for-purpose monitoring and bridging the culture gap between research providers and policy makers by cultivating complementary literacies of what drives and constrains both research and policy.

## Supporting Information

Supporting Information S1 **Grazing management experiment details.**
(DOCX)

Supporting Information S2 **Variables measured and desired Program outcomes.**
(DOC)

Supporting Information S3 **Explanation of the direct monitoring costs.**
(DOCX)

## References

[1] Lindenmayer DB, Cunningham SA, Young A, editors (2012) Land use intensification: Effects on agriculture, biodiversity and ecological processes. Melbourne: CSIRO Publishing. 168 p.

[2] Kerr JT, Deguise I (2004) Habitat loss and the limits to endangered species recovery. Ecol Lett 7: 1163–1169.

[3] Perfecto I, Vandermeer J (2010) The agroecological matrix as alternative to the land-sparing/agriculture intensification model. Proc Natl Acad Sci U S A 107: 5786–5791. 20339080 10.1073/pnas.0905455107 PMC2851926

[4] Cordeiro NJ, Howe HF (2003) Forest fragmentation severs mutualism between seed dispersers and an endemic African tree. Proc Natl Acad Sci U S A 100: 14052–14056. 14614145 10.1073/pnas.2331023100 PMC283544

[5] Hodgson JA, Kunin WE, Thomas CD, Benton TG, Gabriel D (2010) Comparing organic farming and land sparing: optomizing yield and butterfly populations at a landscape scale. Ecol Lett 13: 1358–1367. 20825453 10.1111/j.1461-0248.2010.01528.x

[6] Lindenmayer DB, Bennett AF, Hobbs RJ, editors (2010) Temperate woodland conservation and management. Melbourne: CSIRO Publishing. 400 p.

[7] McIntyre S, McIvor JC, MacLeod ND (2000) Principles for sustainable grazing in eucalypt woodlands: landscape-scale indicators and the search for thresholds. In: Hale P, Petrie A, Moloney D, Sattler P, editors. Management for sustainable ecosystems. Brisbane: University of Queensland. 92–100.

[8] Kleijn D, Sutherland WJ (2003) How effective are European agri-environment schemes in conserving and promoting biodiversity? J Appl Ecol 40: 947–969.

[9] Kleijn D, Berendse F, Smit R, Gilissen N, Smit J, et al (2004) Ecological effectiveness of agri-environment schemes in different agricultural landscapes in the Netherlands. Conserv Biol 18: 775–786.

[10] Henderson B, Norris K (2008) Experiences with market-based instruments for environmental management. Austral J Environ Manage 15: 113–120.

[11] Eigenraam M, Strappazzon L, Lansdell N, Beverly C, Stoneham G (2007) Designing frameworks to deliver unknown information to support market-based instruments. Agric Econ 37: 261–269.

[12] Ferraro PJ (2011) The future of payments for environmental services. Conserv Biol 25: 1134–1138. 22070269 10.1111/j.1523-1739.2011.01791.x

[13] Gibbons JM, Nicholson E, Milner-Gulland EJ, Jones JPG (2011) Should payments for biodiversity conservation br based on action or results? J Appl Ecol 48: 1218–1226.

[14] Zammit C, Attwood S, Burns E (2010) Using markets for woodland conservation on private land: lessons from the policy-research interface. In: Lindenmayer DB, Bennett AF, Hobbs RJ, editors. Temperate woodland conservation and management. Melbourne: CSIRO Publishing. 297–307.

[15] Cardwell M (2010) Rural development in the United Kingdom: continuity and change. Int J Land Law Agr Sci 4: 1–12.

[16] European Commission (2005) Agri-environment measures - Overview on general principles, types of measures, and application. European Commission Directorate General for Agriculture and Rural Development.

[17] Kleijn D, Berendse F, Smit R, Gilissen N (2001) Agri-environment schemes do not effectively protect biodiversity in Dutch agricultural landscapes. Nature 413: 723–725. 11607029 10.1038/35099540

[18] United Kingdom Parliament (2010) HC 611 The outcome of the Comprehensive Spending Review - Supplementary written evidence submitted by the Department for Environment, Food and Rural Affairs (CSR 01A).

[19] Whitfield J (2006) How green was my subsidy? Nature 439: 908–909. 16495965 10.1038/439908a

[20] European Commission (2006) Rural Development policy 2007–2013 - Common monitoring and evaluation framework. European Commission Directorate General for Agriculture and Rural Development.

[21] Bernhardt ES, Likens GE, Hall J RO, Buso DC, Fisher SG, et al (2005) Can’t see the forest for the stream? In-stream processing and terrestrial nitrogen exports. BioScience 55: 219–230.

[22] Brooks SS, Lake PS (2007) River restoration in Victoria, Australia: Change is in the wind and none too soon. Restor Ecol 15: 584–591.

[23] Australian National Audit Office (2007) Audit Report No.21 2007–08 Regional Delivery Model for the Natural Heritage Trust and the National Action Plan for Salinity and Water Quality. Canberra: Australian National Audit Office.

[24] Hajkowicz S (2009) The evolution of Australia's natural resource management programs: Towards improved targeting and evaluation of investments. Land Use Policy 26: 471–478.

[25] Pannell DJ, Roberts AM (2010) Australia's National Action Plan for Salinity and Water Quality: a retrospective assessment. Aust J Agr Res Econ 54: 437–456.

[26] Lindenmayer DB, Likens GE (2010) Effective Ecological Monitoring. Melbourne and London: CSIRO Publishing and Earthscan. 184 p.

[27] Field SA, O'Connor PJ, Tyre AJ, Possingham HP (2007) Making monitoring meaningful. Austral Ecol 32: 485–491.

[28] Muir MJ (2010) Are we measuring conservation effectiveness? Report to Conservation Measures Partnership.Available: www.conservationmeasures.org.

[29] World Bank (1998) Guidelines for monitoring and evaluation of biodiversity projects. Washington, DC: World Bank Global Environmental Division.

[30] Lindenmayer DB, Gibbons P, editors (2012) Biodiversity monitoring in Australia. Melbourne: CSIRO Publishing. 224 p.

[31] Commonwealth of Australia (2009) Environmental Stewardship Strategic Framework. Canberra: Commonwealth of Australia.

[32] OECD (2010) Paying for biodiversity: enhancing the cost-effectiveness of payments for ecosystem services. Paris: OECD.

[33] Ninan KN (2009) Conserving and valuing ecosystem services and biodiversity. London: Earthscan.

[34] Ecker S, Thompson LJ (2010) Participation in the Environmental Stewardship Program Box Gum Grassy Woodland Project: Key Findings and Implications. Canberra: The Australian Bureau of Agricultural and Resource Economics and Sciences (ABARES).

[35] Commonwealth of Australia (2011) Environmental Stewardship Land Manager Reporting Kit - User Guide. Canberra, Australia: Department of Sustainability, Environment, Water, Population and Communities.

[36] Welsh AH, Cunningham RB, Chambers RL (2000) Methodology for estimating the abundance of rare animals: seabird nesting on North East Herald Cay. Biometrics 56: 22–30. 10783773 10.1111/j.0006-341x.2000.00022.x

[37] Cunningham RB, Lindenmayer DB, Crane M, Michael D, McGregor C (2007) Reptile and arboreal marsupial response to replanted vegetation in agricultural landscapes. Ecol Appl 17: 609–619. 17489264 10.1890/05-1892

[38] Lindenmayer DB, Cunningham RB, MacGregor C, Incoll RD (2003) A long-term monitoring study of the population dynamics of arboreal marsupials in the Central Highlands of Victoria. Biol Conserv 110: 161–167.

[39] Lindenmayer DB, Wood J, Montague-Drake R, Michael D, Crane M, et al (2012) Is biodiversity management effective? Cross-sectional relationships between management, bird response and vegetation attributes in an Australian agri-environment scheme. Biol Conserv 152: 62–73.

[40] Kwok ABC, Eldridge DJ, Oliver I (2011) Do landscape health indices reflect arthropd biodiversity status in the eucalypt weoodlands of eastwern Australia? Austral Ecol 36: 800–813.

[41] Montague-Drake R, Lindenmayer DB, Cunningham RB (2009) Habitat determinants of site occupancy for woodland bird species of conservation concern. Biol Conserv 142: 2896–2903.

[42] Ford HA, Walters JR, Cooper CB, Debus SJ, Doerr VA (2009) Extinction debt or habitat change? - Ongoing losses of woodland birds in north-eastern New South Wales, Australia. Biol Conserv 142: 3182–3190.

[43] Martin TG, McIntyre S (2007) Livestock grazing and tree clearing: impacts on birds of woodland, riparian and native pasture habitats. Conserv Biol 21: 504–514. 17391200 10.1111/j.1523-1739.2006.00624.x

[44] Maron M, Lill A (2005) The influence of livestock grazing and weed invasion on habitat use by birds in grassy woodland remnants. Biol Conserv 124: 439–450.

[45] Pyke GH, Recher HF (1983) Censusing Australian birds: a summary of procedures and a scheme for standardisation of data presentation and storage. In: Davies SJ, editor. Methods of censusing birds in Australia. Proceedings of a symposium organised by the Zoology section of the ANZAAS and the Western Australian Group of the Royal Australasian Ornithologists Union. Perth, Australia: Department of Conservation and Environment. 55–63.

[46] Lindenmayer DB, Wood J, MacGregor C (2009) Do observer differences in bird detection significantly influence inferences about environmental impacts? Emu 109: 100–106.

[47] Field SA, Tyre AJ, Possingham HP (2002) Estimating bird species richness: how should repeat surveys be organized in time? Austral Ecol 27: 624–629.

[48] Driscoll D, Milkovits G, Freudenberger D (2000) Impact and use of firewood in Australia. Canberra: CSIRO.

[49] Shine R, Webb JK, Fitzgerald M, Summner J (1998) The impact of bush-rock removal on an endangered snake species *Hoplocephalus bungaroides* (Serpentes: Elapidae). Wildlife Res 25: 285–295.

[50] Lee Y, Nelder JA, Pawitan Y (2006) Generalized linear models with random effects: unified analysis via h-likelihood. Boca Raton: Chapman & Hall/CRC.

[51] Gardner T (2010) Monitoring forest biodiversity. Improving conservation through ecologically responsible management. London: Earthscan.

[52] Spellerberg I (2005) Monitoring ecological change. Cambridge: Cambridge University Press.

[53] Shanley P, Lopez C (2009) Out of the loop: why research rarely reaches policy makers and the public and what can be done. Biotropica 41: 535–544.

[54] McNie EC (2007) Reconciling the supply of scientific information with user demands: an analysis of the problem and review of the literature. Environ Sci Policy 10: 17–38.

[55] Dripps K, Bluml M (2008) Improving the use of science in evidence-based policy: some Victorian experiences in natural resource management. In: Pettit C, Cartwright W, Bishop I, Lowell K, Pullar D et al.., editors. Landscape analyis and visualisation. Berlin: Springer.

[56] Sutherland WJ, Pullin AS, Dolman PM, Knight TM (2004) The need for evidence-based conservation. Trends Ecol Evol 19: 305–308. 16701275 10.1016/j.tree.2004.03.018

[57] Franklin JF, Harmon ME, Swanson FJ (1999) Complementary roles of research and monitoring: lessons from the U.S. LTER Program and Tierra del Fuego. Paper presented to the Symposium; 1999; Guadalajara, Mexico, November 1998.

[58] Carey PD, Pywell RF (2007) An up-to-date cost benefit analysis of English agri-environment schemes: their impact at the landscape scale and the cost of adequate monitoring. In: Bunce RGH, Jongman R, Hojas L, Weel S, editors. 25 Years of Landscape Ecology: Scientific Principles in Practice. The Netherlands: International Association for Landscape Ecology. 70–71.

[59] Bernhardt ES, Palmer MA, Allan JD (2005) Synthesizing US river restoration projects. Science 308: 636–637. 15860611 10.1126/science.1109769

[60] Whittingham MJ (2007) Will agri-environment schemes deliver substantial biodiversity gain, and if not why not? J Appl Ecol 44: 1–5.

[61] Kleijn D, Baquero RA, Clough Y, Diaz M, De Estaban J, et al (2006) Mixed biodiversity benefits of agri-environment schemes in five European countries. Ecol Lett 9: 243–254. 16958888 10.1111/j.1461-0248.2005.00869.x

[62] Fuentes-Montemayor E, Goulson D, Park KJ (2011) Pipistrelle bats and their prey do not benefit from four widely applied agri-environment management prescriptions. Biol Conserv 144: 2233–2246.

[63] Perkins AJ, Maggs HE, Watson A, Wilson JD (2011) Adaptive management and targeting of agri-environment schemes does benfit biodiversity: a case study of the corn bunting Emberiza calandra. J Appl Ecol 48: 514–522.

[64] Roth T, Amrhein V, Peter B, Weber D (2008) A Swiss agri-environment scheme effectively enhances species richness for some taxa over time. Agr Ecosyst Environ 125: 167–172.

[65] Lindenmayer DB, Archer S, Barton P, Bond S, Crane M, et al.. (2011) What makes a good farm for wildlife? Melbourne: CSIRO Publishing. 160 p.

[66] Munro N, Lindenmayer DB (2011) Planting for wildlife: A practical guide to restoring native woodlands. Melbourne: CSIRO Publishing.

[67] Gibbons P, Zammit C, Youngentob K, Possingham HP, Lindenmayer DB, et al (2008) Some practical suggestions for improving engagement between researchers and policy-makers in natural resource management. Ecol Manage Restor 9: 182–186.

[68] Lawton J (2007) Ecology, politics and policy. J Appl Ecol 44: 465–474.

[69] Rudd MA (2011) How research-prioritization exercises affect conservation policy. Conserv Biol 25: 860–866. 21790784 10.1111/j.1523-1739.2011.01712.x

[70] Alberts A (2011) Science adapters wanted. Science 334: 1031. 22116841 10.1126/science.1216650

[71] Cheng D, Claessens M, Gascoigne T, Metcalfe J, Schiele B, et al.. (2008) Communicating science in social contexts Berlin: Springer Science.

