# Supplementary material for: A Novel and Cost-Effective Monitoring Approach for Outcomes in an Australian Biodiversity Conservation Incentive Program
Source: PLoS One. 2012 Dec 6;7(12):e50872. doi: 10.1371/journal.pone.0050872 (PMC3516526; doi:10.1371/journal.pone.0050872)
Supplement: Supporting Information S1 — Grazing management experiment details. (DOCX) [file pone.0050872.s001.docx]

**Supporting Information S1: Grazing management experiment**

*A grazing management regime experiment allied to the objective and desired outcomes of the Environmental Stewardship Program’s Box Gum Grassy Woodland Project*

The ecological monitoring aims to quantify temporal changes in vegetation condition and fauna populations, and explicitly link changes in vegetation condition over time with changes in bird and reptile biota. However, one of the key practices central to the Box Gum Grassy Woodland Project is the management of domestic livestock grazing, particularly because it is widely considered to be a key driver of woodland condition (reviewed by [6,51]).

Past research suggests that grazing intensity can be managed in ways that retain high levels of plant biodiversity in Australian temperate woodlands [52]. Accordingly, we designed a grazing experiment to inform the Program about successful (and also unsuccessful) biodiversity-focused grazing regimes. Thus, while the main objective of the Project monitoring is to determine **if** change has occurred, the experiment attempts to determine **why** such change (if any) has occurred through explicitly quantifying the impacts of grazing regimes on vegetation condition, birds and reptiles.

The grazing experiment encompasses 30 farms. There are ten farms in each of three broad categories (a small subset of which are not Project farms, which was necessary to increase replication). The farm categories were:

- **Farm Type 1**: “Business as usual” production farms that have been subject to conventional set-stocking grazing regimes for the past 10 years or longer.
- **Farm Type 2**: “Holistic farms” that for ≥10 years have employed holistic, short-duration, high-intensity grazing.
- **Farm Type 3**: “Transition farms” in which grazing practices have changed in the past three years from set-stocking to holistic grazing regimes.

On each farm, we established three matched sites to which we applied grazing treatments. On one site, domestic livestock grazing is entirely excluded through fencing; on a second site, the Project grazing regime is implemented (no grazing from September to February, and grazing from March to August if maintaining vegetative groundcover of >70% across the patch); and on a third site, ‘business as usual’ grazing is applied. The response variables in the experiment are the same as in the ecological monitoring; *viz*: vegetation condition and the presence and abundance of reptiles and birds.

The results of the experiment will be provided to the Australian Government in the annual report on the monitoring, including any evidence-based recommendations for alterations to the prescribed Project grazing regime. Alterations may subsequently be negotiated into land manager contracts.
