# Supplementary material for: A Novel and Cost-Effective Monitoring Approach for Outcomes in an Australian Biodiversity Conservation Incentive Program
Source: PLoS One. 2012 Dec 6;7(12):e50872. doi: 10.1371/journal.pone.0050872 (PMC3516526; doi:10.1371/journal.pone.0050872)
Supplement: Supporting Information S2 — Variables measured and desired Program outcomes. (DOC) [file pone.0050872.s002.doc]

**Supporting Information S2: Variables measured and desired Program outcomes**

*Links made between each biophysical measure in the monitoring protocol and the desired policy outcomes as stated in the Environmental Stewardship Program’s* Strategic Framework.

| **Habitat layer** | **Measure** | **How relates to Program desired outcomes from Strategic Framework** |
| --- | --- | --- |
| **Ground cover** | **Percentage cover of:** |  |
|  | Native grasses | Improved condition; improved habitat |
|  | Native forbs/herbs | Improved condition; improved habitat |
|  | Native sub-shrubs (<1m) | Improved condition; improved habitat |
|  | Total native cover (sum of above) | Improved condition; improved habitat |
|  | Bare ground | Improved condition; improved function |
|  | Organic litter | Improved habitat; improved function |
|  | Fallen dead wood | Improved habitat |
|  | Cryptogams | Improved condition; improved function |
|  | Annual exotic grasses | Improved condition; improved habitat |
|  | Perennial exotic grasses | Improved condition; improved habitat |
|  | Exotic plants (other) | Improved condition; improved habitat |
|  | Total exotic plant cover (sum of above) | Improved condition; improved habitat |
|  | Ratio of native and exotic plant cover | Improved condition; improved habitat |
|  | **Counts** |  |
|  | Native plant species richness | Improved condition; improved habitat; improved function; improved viability |
|  | Native plant species list | Improved condition; improved habitat; improved function; improved viability |
|  | Native plant functional groups/lifeforms | Improved condition; improved habitat; improved function; improved viability |
|  | Tree/shrub recruitment | Improved condition; improved habitat; improved function; improved viability |
| **Midstory  (1-2 m)** | **Percentage cover of:** |  |
|  | Native mid-storey cover | Improved condition; improved habitat |
|  | Exotic mid-storey cover | Improved condition; improved habitat |
|  | **Counts** |  |
|  | Native mid-storey richness | Improved condition; improved habitat |
|  | Native mid-storey recruitment | Improved condition; improved habitat; improved function; improved viability |
| **Overstory** | **Percentage cover of:** |  |
|  | Native overstorey (projected foliage cover) | Improved condition; improved habitat |
|  | Canopy foliage health/condition | Improved condition; improved habitat; improved function; improved viability |
|  | Native overstorey age class distribution | Improved condition; improved habitat; improved function; improved viability |
|  | **Counts** |  |
|  | Native overstorey stem density | Improved condition; improved habitat |
|  | Native overstorey richness | Improved condition; improved habitat; improved function; improved viability |
|  | Hollow bearing trees | Improved habitat |
|  | Canopy connectivity | Improved habitat |
|  | Mistletoe clumps | Improved habitat; improved condition |
|  | Evidence of stock (faeces, tracks, camps) | Improved condition; improved habitat |
| **Threats** | Evidence of macropods | Improved condition; improved habitat |
|  | Evidence of rabbits/hares | Improved condition; improved habitat |
|  | Evidence of erosion | Improved condition; improved function |
|  | E.g. Fire, ripping, ploughing. | Improved condition; improved function; enduring land manager attitudes |
|  | Soil erosion | Improved condition; improved function; enduring land manager attitudes |
| **Fauna** | Bird species richness | Improved habitat, improvements in protection of nationally endangered species, increased viability |
|  | Bird assemblage composition | Improved habitat, improvements in protection of nationally endangered species, increased viability |
|  | Reptile species richness | Improved habitat, improvements in protection of nationally endangered species, increased viability |
|  | Reptile assemblage composition | Improved habitat, improvements in protection of nationally endangered species, increased viability |
