# Supplementary material for: A Novel and Cost-Effective Monitoring Approach for Outcomes in an Australian Biodiversity Conservation Incentive Program
Source: PLoS One. 2012 Dec 6;7(12):e50872. doi: 10.1371/journal.pone.0050872 (PMC3516526; doi:10.1371/journal.pone.0050872)
Supplement: Supporting Information S3 — Explanation of the direct monitoring costs. (DOCX) [file pone.0050872.s003.docx]

**Supporting Information S3: Explanation of the direct monitoring costs**

Two full-time field staff are required to run the ecological monitoring for the Box Gum Grassy Woodland Project. These staff are needed because of the large and often isolated area over which field sites are located, and the large amount of field survey work that needs to be completed. In addition, to meet occupational health and safety rules set by The Australian National University, staff must complete all survey work in pairs. In Table 1, the salaries and costs for these two staff are assigned to the project management section of the budget when they are not involved in formal field monitoring surveys. During this time they are involved in project management tasks such as liaising with landholders and staff from Catchment Management Authorities, and quantifying responses of biota to the various management treatments in the grazing experiment. Other project management costs include the purchase of two 4WD vehicles, and a mandatory university overhead of 8% of the total funding.

The design of the ecological monitoring was enabled by a detailed assessment of the relevant literature, e.g. [53], and consideration of the merits of alternative sampling and statistical designs. In addition, a pilot study was conducted to test field protocols and collect data for subsequent pilot statistical analyses that were conducted prior to the establishment of field sites to assess the power of the proposed design. The funds in this part of budget covered the costs of a professional statistical scientist to conduct these various analyses.

The site set-up and maintenance component of the budget for 2009-2010 included detailed reconnaissance and landholder liaison to locate stewardship sites and, wherever possible, to identify a suitable matched control site. This was followed by the deployment of survey infrastructure on all 268 field sites. This included 820 steel pickets used as permanent site markers and the deployment of artificial substrates for reptile surveys at each site totaling ~ 2,500 roof tiles, 1,700 sheets of corrugated iron and 1,700 wooden sleepers. The equivalent of one person day of field work was required to establish both the stewardship and control sites on each of the 153 farms.

Following the establishment of the permanent field sites, a major time component of the ecological monitoring is the repeated surveys of vegetation condition, birds and reptiles. The costs of the surveys included the salaries of the two full-time staff for the duration of the time they were in the field. The survey component of the budget also includes the salaries and other costs of additional experienced and technically-expert staff recruited for the ecological monitoring to ensure that surveys across the entire study were completed quickly (typically within ~four weeks). This was essential to avoid confounding with factors like seasonal effects. The use of highly trained additional field staff was critical to reduce the potential problems of observer heterogeneity in gathering data on the presence and abundance of plant and animal taxa [46,54].

We calculated the amount of time in person days to complete each of these major surveys. We have accounted for travelling time between farms and sites within each farm, and added 10 days to each survey type to allow for the contingencies of bad weather, access difficulties or delays. For vegetation surveys, it required an average of 2.77 hours to complete work on both the stewardship and control site on a given farm; reptile surveys and bird surveys each took 1.21 hours to complete per farm. With travel time and contingencies, this corresponds to 150 person days for a full vegetation survey, 56 person days for a full reptile survey, and 133 person days for the bird surveys (two per year for ~66 days each).

The budget includes a line item for statistics. This was to cover the costs of an expert, professional statistical scientist to complete analyses of datasets on vegetation, birds and reptiles (e.g. Figures 3, 4, 5).
